# Surgical Approaches in Odontogenic Orbital Cellulitis (OOC): Our Experience and Review of Literature

**DOI:** 10.1007/s12070-021-02576-y

**Published:** 2021-05-02

**Authors:** Romano Antonio, Troise Stefania, Committeri Umberto, Arena Antonio, Giovanni Dell’Aversana Orabona, Seidita Francesco, Bonavolontà Paola, Iaconetta Giorgio, Califano Lugi

**Affiliations:** 1 Department of Maxillofacial Surgery, Federico II University of Naples, Via Pansini, 5, 80131 Naples, Italy; 2 Neurosurgery Department, University of Salerno, Via Giovanni Paolo II, 132, 84084 Fisciano, Salerno, Italy; 3 Department of Neuroscience and Reproductive and Odontostomatological Sciences, Maxillofacial Surgery, School of Medicine, University of Naples “Federico II”, Via Sergio Pansini, 5, 80131 Naples, Italy

**Keywords:** Odontogenic orbital cellulitis, Surgical approach, Dental extraction, Early diagnosis

## Abstract

*Aims* Odontogenic orbital cellulitis represents a complication of root infections of upper pre-molars and molars. The severity depends on the orbital structure involved. The treatment is based on antibiotic therapy associated or not to surgery. Through the presentation of three cases and a review of literature, we purpose as aim of our study to underline the necessity of a timely diagnosis and to provide the correct surgical approach in each different types and stages of orbital infections. *Methods* We present three patients that were affected by dental infection evolved in orbital cellulitis. In two cases the disease was solved with the extraction of infected tooth and a surgical endoscopic drainage of the abscess through antrostomy of maxillary sinus. In the third patient the disease had already induced a bulbar perforation and endophthalmitis, so an orbit evisceration was necessary. *Results* Review of literature showed that the standard treatment of orbital cellulitis is the transnasal approach associated or not by a transoral and/or transcutaneous procedure depending on the stage of the disease and on the causes. In our 3 cases these indications were followed without relapses of the disease*. Conclusion* An early diagnosis is mandatory in odontogenic orbital cellulitis specially to avoid serious complications. Surgical treatment can be simple and effective mostly in early-stage infection: it is based on extraction of infected tooth and on the drainage of abscess. Surgical approach consists in transnasal procedure flanked or not by transoral and transcutaneous procedures based on the stage of the infection considering involved structures.

## Introduction

Orbital cellulitis (OC) and subperiosteal abscess (SPA) represent an inflammatory disorder that occur to orbital tissues. These conditions admit many causes, mainly: trauma, orbital foreign body, dacryocystitis and endophthalmitis [1, 2].

Approximately not more 2–5% of cases of OC are odontogenic (OOC). In this rare case the OC is configured as a complication relative to a root infection of upper pre-molars and molars. The root would be directly in contact with the maxillary sinus floor mucosae, providing a passage of microorganisms. In this way, the odontogenic bacterial infection spread to the orbit through the paranasal sinuses. In the most serious cases, a central spread of the infection that induces then an intracranial sequela such as cavernous sinus thrombosis, meningitis, and encephalitis is possible [3].

Microbial cultures of orbital cellulitis typically show polymicrobial infections, including anaerobic Bacteroides species, streptococcus, in particularly Streptococcus Pyogenes and Pneumoniae, Prevotella, Fusobacterium and, in children, Hemophilus influenzae. Organisms most reported include Staphylococcus Aureus and alfa hemolytic Streptococcus [3, 4].

Clinically, orbital cellulitis begins with general and non-specific signs and symptoms such as severe eyelid redness and edema, ptosis, conjunctival chemosis, discharge, erythema of periorbital tissues, and periocular pain or pain due to eye movements. As soon as the infection progresses, there are specific signs such as proptosis and globe displacement, dystopia, decreased vision, impaired color vision, and limited ocular motility. In the cases of odontogenic origin, there are symptoms of dental involvement, such as face and cheek swelling, teeth and face pain, bad breath and dental hypersensitivity [5].

In radiological imaging the first step is represented by orthopantomography (OPG) that evaluates the involvement of the dental elements. II level of evaluation is Computerized Tomography (CT) helpful to identify the sinuses involvement, orbital abscesses, and any cerebral complications. Magnetic Resonance Imaging (MRI) can be useful in evaluation of the retro-maxillary soft tissues and cavernous sinus.

Thanks to the anatomical district of “orbital septum”, which is configured as the most anterior extension of the periosteum side of the orbit, the OC are classified in: pre- and post- septal.

Pre-septal inflammation shows eyelid edema in the absence of orbital signs. It is caused by a direct inoculation of the pathogenic microbes through a skin infection. Swelling of the eyelid remains anterior to the orbital septum and it is the result of the impeded venous and lymphatic drainage.

In post-septal inflammation, the orbital tissue is diffusely infiltrated by inflammatory cells and bacteria. According to the posterior orbit and cerebral involvement, the degree of proptosis, the visual impairment and other complications change [6, 7].

In 1970, Chandler et al. classified orbital cellulitis and their complications based on localization and severity of the infection [4] (Table 1).

**Table 1:** Chandler classification, based on localization and severity of the infection, permits to value orbital cellulitis and their possible complications

Chandler’s Classification	
Group 1: Preseptal cellulitis	The inflammatory process is limited anteriorly to the orbital septum and does not invade the infraorbital structures
Group 2: Orbital cellulitis (OC)	The orbital tissues are diffusely affected
Group 3: Subperiosteal abscess (SPA)	Purulent material collects in periorbital tissues, between the bony walls of the orbit and the periorbita
Group 4: Intraorbital abscess (IOA)	There is a purulent collection inside the orbit
Group 5: Cavernous sinus thrombosis	There is an extension of orbital inflammation into the cavernous sinus that can lead to involvement of the third, fifth, and sixth cranial nerves

Historically, this classification based on the severity of clinical and radiological images has been useful in order to differentiate the affected structures and, therefore, the treatment. Pre-septal cellulitis responds to antibiotics alone while Orbital cellulitis or sub-periosteal abscess require a surgical procedure, from the incision and drainage of the periapical abscess or tooth extraction, to endoscopic sinus surgery until to an evisceration surgery. Currently, endoscopy is considered a valid instrument that respects the functionality of the paranasal sinuses, without altering the anatomy of the ciliated epithelium inside [8].

The aim of our article is to point up the importance of early diagnosis of the disease, the prompt identification of the odontogenic origin and the use of a correct surgical algorithm, to quickly remove the cause of the infection. We present three cases in different stages of orbital infection, and we attempt to choose, through a brief review of the literature, the most appropriate surgical approach, based on the different stages of the disease.

## Material and Methods

This study was granted an ethical exemption by Federico II University, Naples, Italy, due to its retrospective nature. We present three cases of patients affected by odontogenic orbital cellulitis, admitted to our Department of Maxillo-Facial Surgery between November 2017 and July 2018. We also analyzed the most recent retrospective studies, on the topic of surgical approaches of odontogenic orbital cellulitis, in the literature from 2001 to 2019 with more than 10 cases described and we realized a brief review.

## Case Series

### Case 1, Group 2: Orbital Cellulitis (OC)

A 49-year-old man was hospitalized in our Department of Maxillo-Facial Surgery of the Federico II University of Naples on April 2018. For four days, the patient had complained a swelling of the lower eyelid in the right eye. At the clinical examination, in the right eye there was evidence of conjunctival chemosis, hyperemia, exophthalmos, and dystopia. Ocular motility and eye reflexes were preserved. The left eye did not show signs or symptoms of infection.

**Fig. 1:**
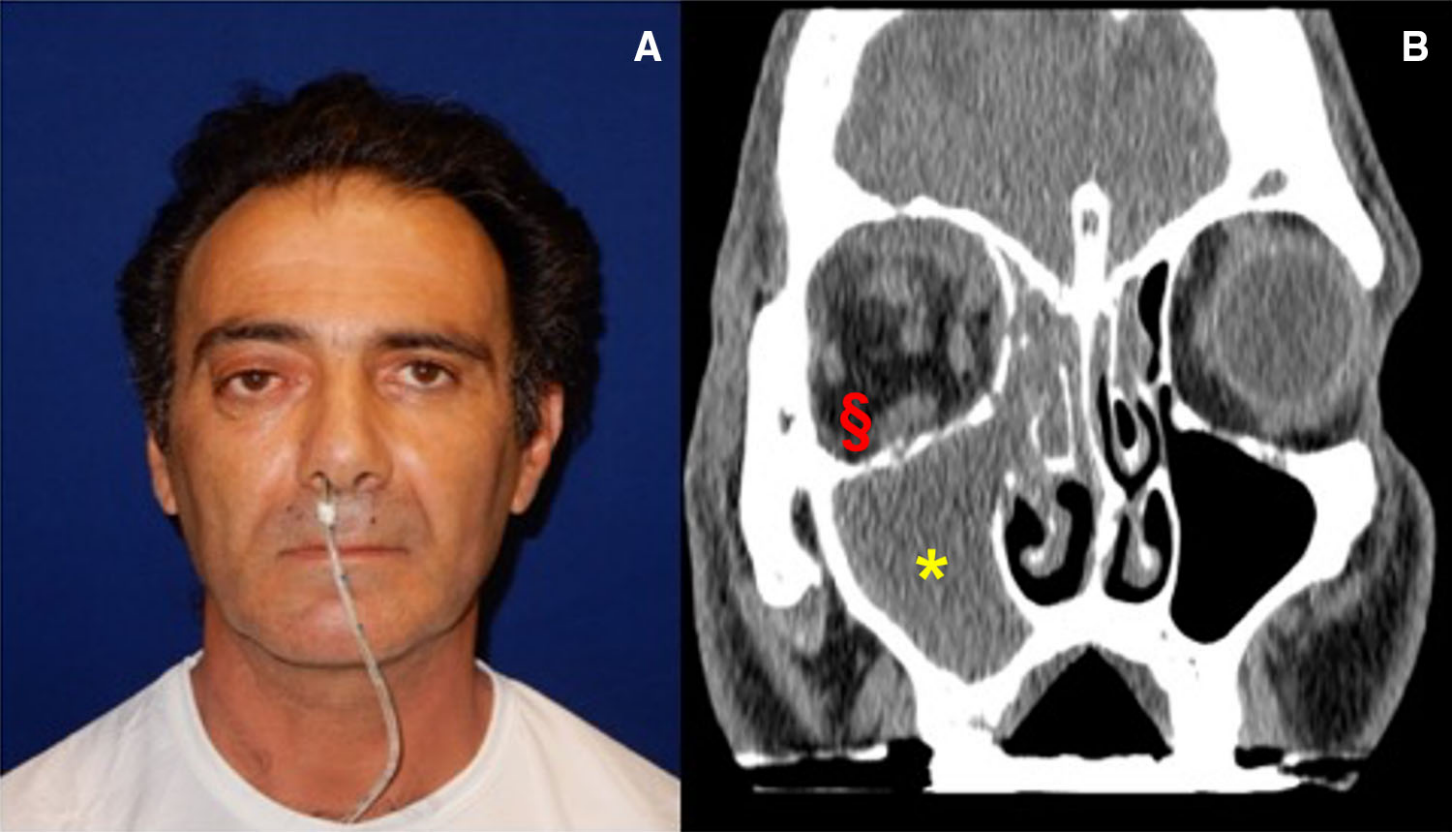
Case 1: Presurgical frontal photo: limitation movement of right eye not consensual with the left one (**a**) and CT Imaging in coronal section: maxillary sinus occupied by abscess (*), periorbital inflammatory degeneration of tissues (§) (**b**)

Computed Tomography (CT) of paranasal sinuses and orbit, revealed that right maxillary sinus, ipsilateral nasal fossa, ethmoid cells, and medial region of the orbit were occupied by inflammatory tissue. Soft tissue edema was also present in the orbital section. The oral examination revealed the presence of a caries of the 1.6 dental element; the orthopantomography (OPT) confirmed the caries and revealed a root involvement, as well as peri-radicular tissues. A nasal fibroscopy revealed purulent inflammation in the right inferior, middle, and superior meatus, and in right uncinate process. (Fig. 1).

Based on the clinical and radiological characteristics, the patient belonged to the second group of the Chandler’s classification with an odontogenic infection; so, our plane was combined antibiotic and surgical treatment.

The patient was treated, at first, with empiric antibiotic therapy (Ceftriaxone 2 g iv 1/die, Levofloxacin 500 mg Intravenous (IV) 1/die, Metronidazole 500 mg IV 1/die and topic Tobramycin). Then the cultural and blood tests revealed a polymicrobial infection with predominance of Bacterioides species, so after consulting with Infectious Disease Department, the therapy dosed was: Vancomycin 500 mg IV four times in die (Q.I.D.), Levofloxacin 500 mg IV twice a day (B.I.D.), Metronidazole 500 mg IV every day (Q.D.) and topical Tobramycin. A week later, the patient showed a clinical improvement and underwent surgical procedure based on a 1.6 dental element extraction, and endoscopic antrostomy of the right maxillary sinus ostium and anterior ethmoidectomy, with drainage of the purulent material.

**Fig. 2:**
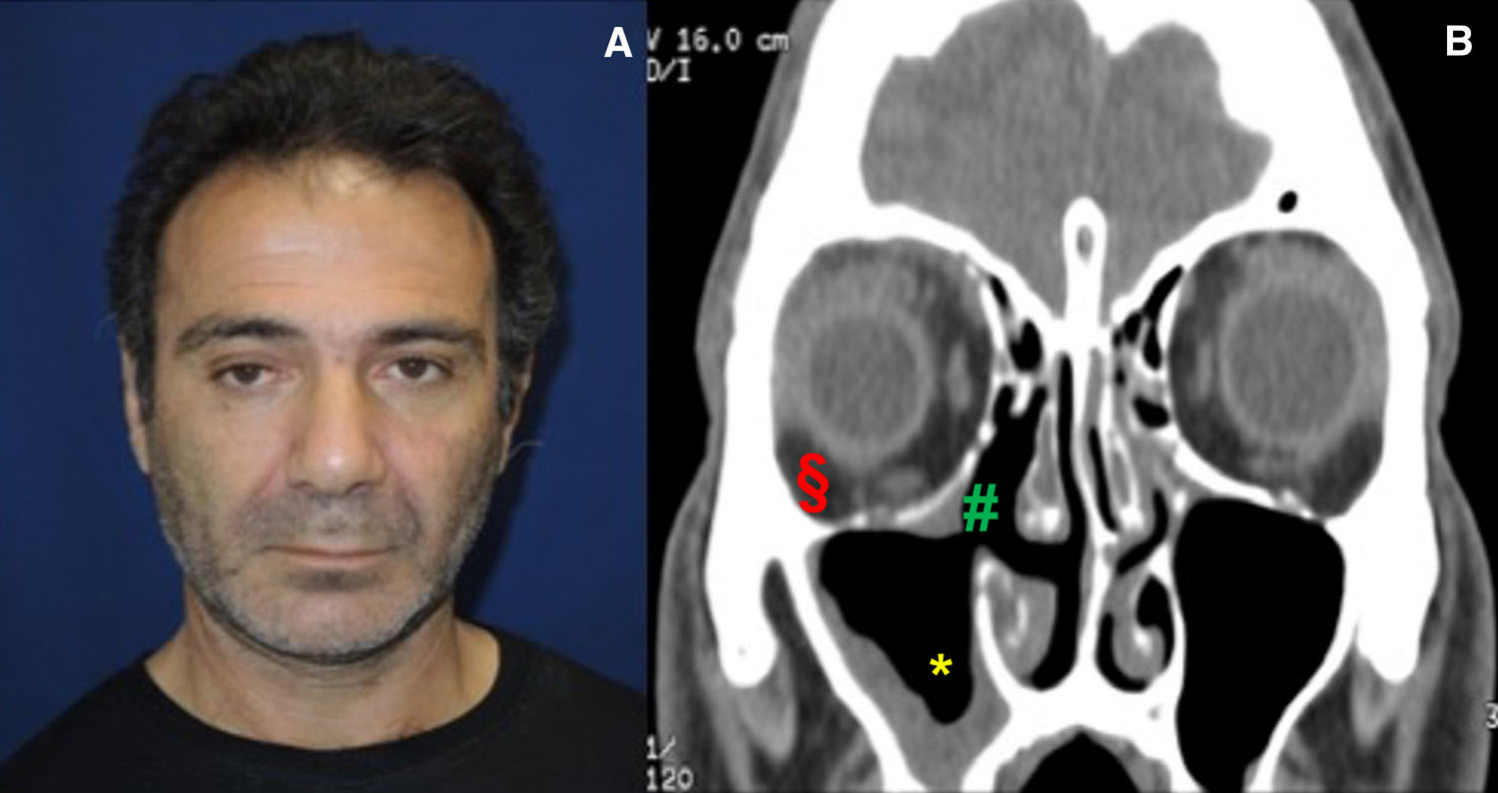
Case 1: Postsurgical photo of patient in frontal section: rehabilitation of consensual extrinsic motility of the eyes (**a**) and CT Imaging in coronal section: maxillary sinus empty of abscess secretions (*), periorbital edema reduction (§), site of antrostomy (#) (**b**)

The patient was discharged with antibiotic oral therapy, steroids, saline solution nasal washes, and oral hygiene advices. During follow-up examination (one week, one month, six months and one year), the endoscopic and CT controls showed reduction of inflammation and the patient had no relapse of the infection (Fig. 2.)

### Case 2, Group 3: Subperiosteal Abscess (SPA)

A 17-year-old male was hospitalized on July 2018 in the Orbital Surgery Unit of the Ophthalmologic Department of the Federico II University of Naples; one week before the patient complained swelling on the upper and lower eyelids in the left eye. At clinical examination there was evidence of conjunctival chemosis, exophthalmos and dystopia. Ocular and lower eyelid itch were referred and limitation in ocular motility were observed. The eye reflexes were preserved. The right eye did not show signs or symptoms of infection.

Magnetic Resonance Imaging (MRI) of paranasal sinuses and orbit revealed the presence of inflammatory tissue in frontal-ethmoidal-maxillary sinuses that induced an erosion of medial wall of the orbit, and dislocation of medial rectus muscle. Soft tissue edema was also present in the orbit. Coronal slices showed a subperiosteal abscess, all along the medial wall of the left orbit and orbital cellulitis. The oral examination revealed the presence of a solution of continuity of the enamel on the 2.6 dental element. The panoramic radiograph confirmed the presence of caries and showed a periapical lesion of 3 mm. Nasal fibroscopy revealed purulent inflammation in the left inferior and middle meatus.

**Fig. 3:**
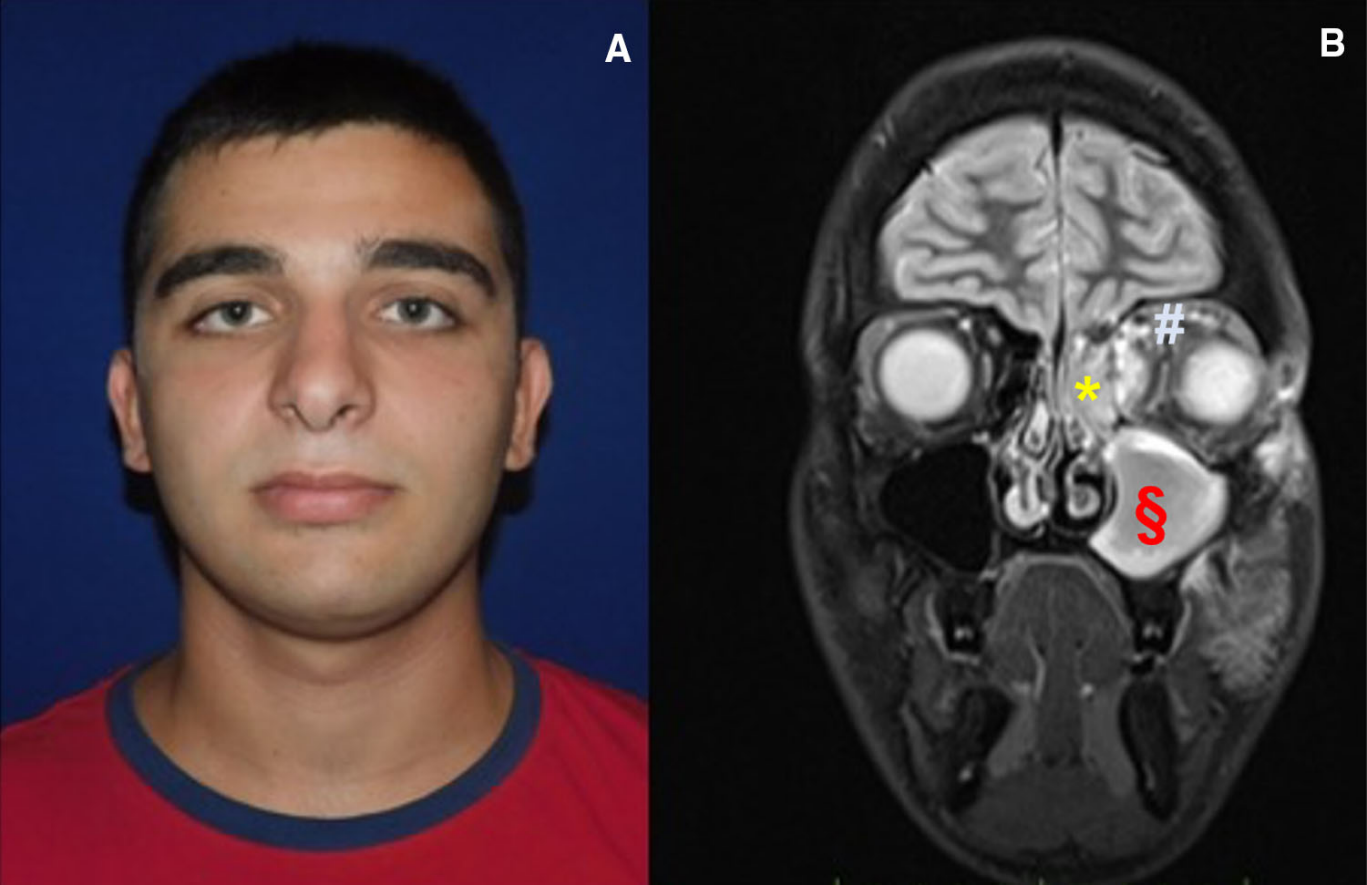
Case 2: Presurgical photo of patient in frontal section: swelling from abscess collection to supero-medial localization in the left orbit (**a**) and MRI Imaging in coronal section: maxillary sinus occupied by abscess (*), periorbital inflammatory degeneration of tissues (§), (**b**)

Based on the clinical and radiological characteristics, the patient belonged to the third group of the Chandler’s classification, hence our plane was combined antibiotic and surgical treatment (Fig. 3).

The patient immediately initiated an empiric antibiotic therapy (Vancomycin 500 mg iv Q.I.D., Levofloxacin 500 mg IV B.I.D., Metronidazole 500 mg IV Q.D. and topical Tobramycin). Cultural blood tests revealed polymicrobial infection with presence of Staphylococcus aureus and Infectious Disease Department confirmed the therapy. After a week the patient showed a clinical improvement, therefore he underwent 2.6 dental element extraction followed by endoscopic antrostomy of the left maxillary sinus ostium, anterior and posterior ethmoidectomy, with drainage of the purulent material according to DRAF 1 technique (it includes the resection of the Uncinate process in addition to the resection of medial lamella of the Agger Nasi cell and the anterior wall of the ethmoid bulla) and a superior, middle and inferior turbinoplasty.

**Fig. 4:**
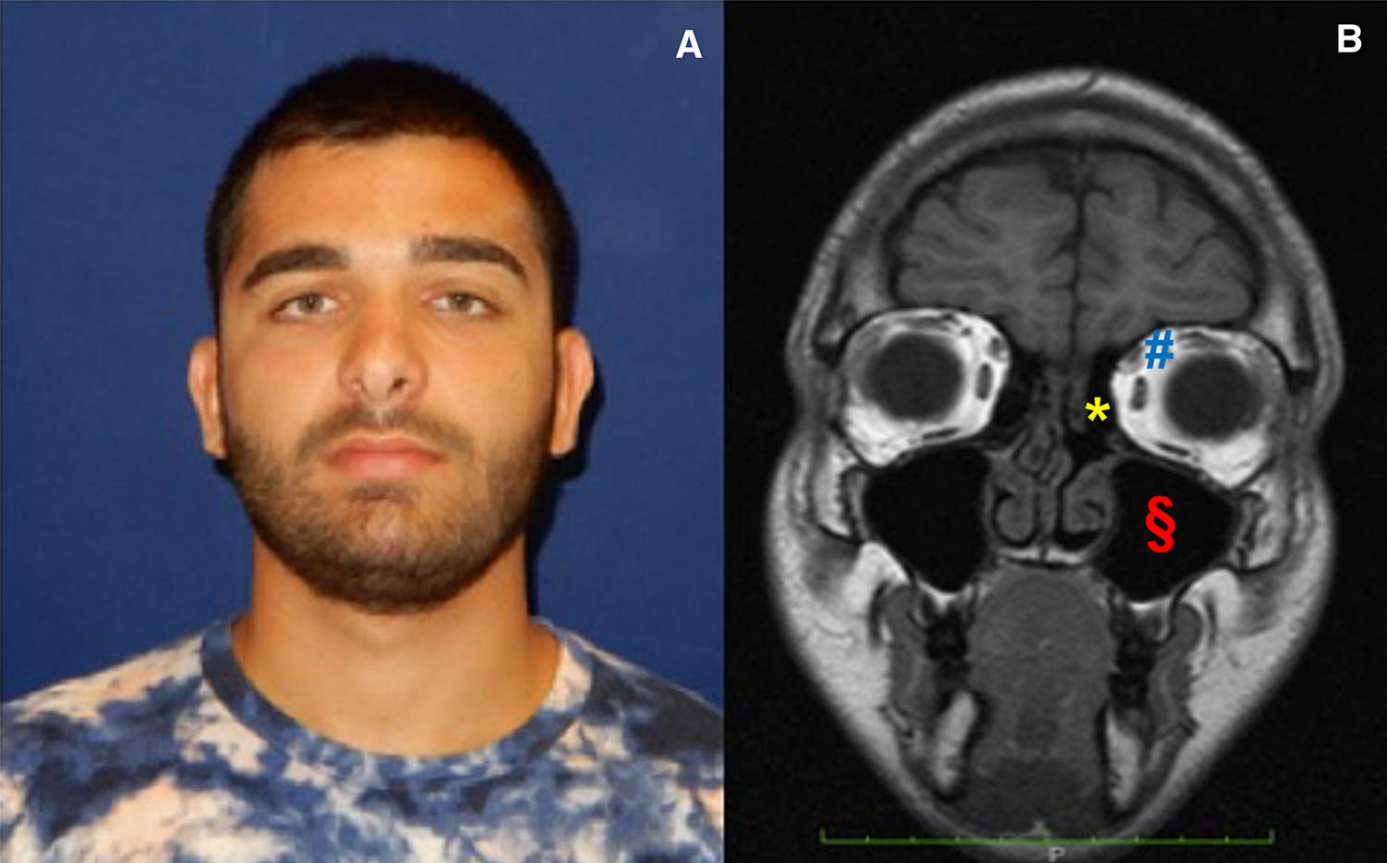
Case 2: Postsurgical photo of patient in frontal section: resolution of swelling induced by abscess in supero-medial section of the left orbit (**a**) and MRI Imaging in coronal section: maxillary sinus totally empty (§), rehabilitation of periorbital tissues (#), ethmoid site drained of purulent secretions (*) (**b**)

The patient was discharged with antibiotic therapy, steroids, saline solution nasal washes, and oral hygiene advices. During follow-up examination (one week, one month, six months and one year) the endoscopic and MRI controls showed reduction of inflammation and the patient had no relapse of the infection (Fig. 4).

### Case 3, Group 4: Infraorbital Abscess (IOA)

A 50-year-old woman was referred to the Department of Maxillo-Facial Surgery of the University Federico II of Naples on November 2017 [9].

**Fig. 5:**
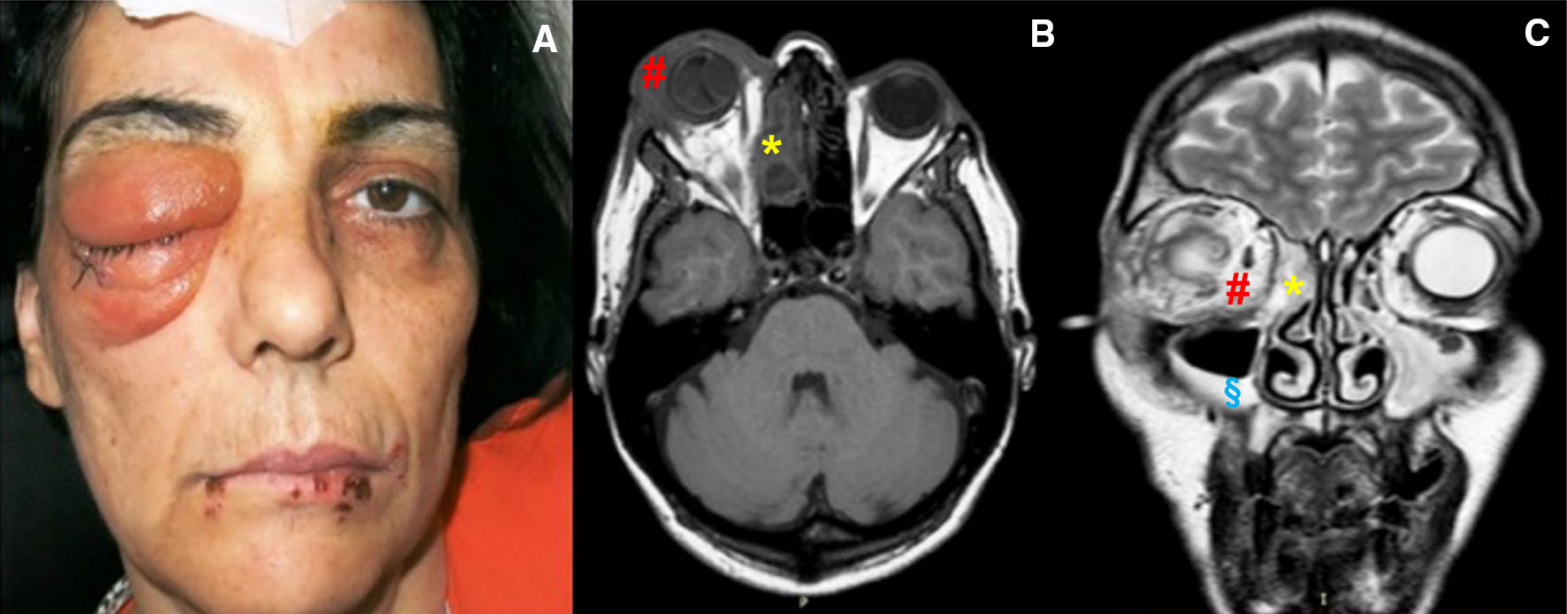
Case 3: Presurgical photo of patient in frontal section (**a**) and MRI Axial section (**b**) and MRI Coronal section (**c**): ethmoid abscess (*), hyper-haemic maxillary sinus mucosa (§), orbital abscess (#)

She was previously referred to another hospital with a history of rapidly progressive inflammation in the right orbit, not responsive to antibiotic and corticosteroid therapy. At hospitalization, she had bulbar perforation and fever, but hemodynamically stable. The clinical examination of the right orbit revealed proptosis, superior and inferior eyelid swelling, and erythema. There was a complete opening limitation of eye. The eye was hypotonic and partially covered by a conjunctival patch. The conjunctiva was hyperemic and chemotic, and there was also severe corneal edema. Anterior chamber, lens, vitreous and retina were not assessable, due to corneal opacity. Visual acuity in the right eye was only scanty light perception. The left eye was normal, with no signs or symptoms of infection. MRI of head and neck, sinuses and orbit, revealed an important right infraorbital abscess with optical nerve involvement, displacement of extrinsic muscles, and right maxillary sinus empyema, extending to right anterior and posterior ethmoidal cells. The oral examination revealed very scanty oral hygiene, with tartar and dental plaque, dental caries and diffuse periodontal disease the orthopantomography (OPT) demonstrated horizontal and vertical bone loss, periodontal disease and dental caries. These findings were compatible with an odontogenic infection. According to the clinical and radiological characteristics, the patient belonged to the fourth group of the Chandler’s classification, and our plane was combined antibiotic and surgical treatment. (Fig. 5).

The patient was immediately treated with empiric antibiotic therapy (Ceftriaxone 2 g IV 1/die, Levofloxacin 500 mg IV B.I.D., Metronidazole 500 mg iv Q.D. and topic Tobramycin). A sample of orbital fluid was obtained and analyzed. The cultural exam revealed Propionibacterium spp and Bacteroides spp. With the agreement of the Infectious Disease Department, the therapy was modified to Vancomycin 500 mg IV Q.I.D., Levofloxacin 500 mg IV B.I.D., Metronidazole 500 mg IV Q.D. and topical Tobramycin. The patient showed a clinical improvement, nonetheless the eye condition did not enhance. For this reason, an urgent surgical removal of the eyeball was performed, in order to avoid the high risk of intracranial complications. After eye surgery, the patient underwent a complete dental extraction.

**Fig. 6:**
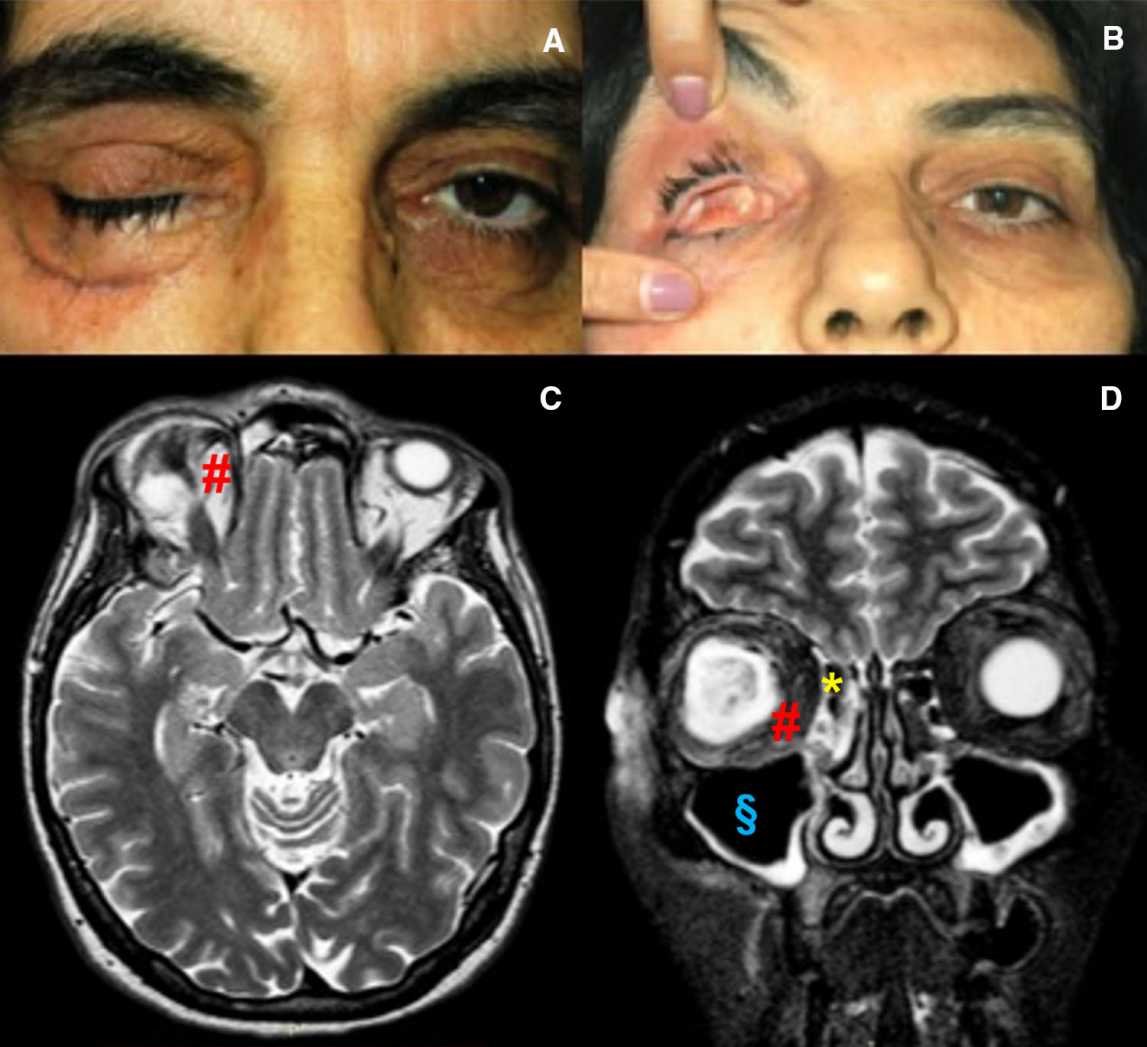
Case 3: Postsurgical photo of patient in frontal section: closed eyelid (**a**), open eyelid showing orbit evisceration (**b**), MRI in Axial section (**c**) and MRI Coronal section (**d**): ethmoid sinus drained of purulent secretions (*), maxillary sinus totally empty (§), resolution of the orbital abscess (#)

The patient was discharged with antibiotic oral therapy, steroids, saline solution nasal washes, and oral hygiene advices. During follow-up examination (one week, one month, six months, and one year) the endoscopic and MRI controls, showed reduction of inflammation and the patient had no relapse of the infection in the right orbit, paranasal sinuses and oral cavity. (Fig. 6.)

## Discussion

Orbital cellulitis commonly occurs (84% of cases) from bacterial infections; other causes are periorbital trauma, periocular infections, orbital surgery, ascending thrombophlebitis. Many reasons predispose the tissues of the orbital region to serious consequences when an infection spreads: in particular the “closed box” anatomy of the orbit and the poor lymphatic drainage cause the stagnation of access and its extension in the pre-septal and periorbital area.

Odontogenic orbital cellulitis (OOC) is uncommon, but it is a potential cause of severe complications. OOC approximately represent 2–5% of cases of orbital cellulitis: it usually occurs when the integrity of the Schneiderian’s membrane of paranasal sinuses is interrupted by odontogenic disease or after dental procedures, such as implant placement, tooth extraction, caries, delayed eruption, endodontic procedures, and sinus lifts [5]. Felisati et al. [10] proposed a division in categories according to initial dental treatment: group I, pre-implant treatment; group II, implantology treatment; and group III, dental treatment. According to these categories, the Authors suggested a different approach based on transoral procedure and/or endoscopic surgery.

There are several ways through which an odontogenic infection can spread to the orbit. Root apices are anatomically proximal to adjacent muscle, connective tissue, and sinuses. First, the infection can pass through the maxillary or ethmoidal sinuses because of bone erosions or dehiscence, and it can spread in the orbital floor, the infraorbital neuromuscular canal or in the lamina papyracea. Second, the infection can enter the posterior orbit, directly through the inferior orbital fissure after the penetration of the pterygopalatine and infratemporal fossae. Third, the valveless nature of the venous system of the orbit facilitates the dissemination of the infection. Last, the odontogenic infection can complicate with the perforation of eyelid reaching therefore the pre-septal space and subsequently the orbit [11, 12].

Medical management focuses primarily on aggressive antibiotic therapy [13]. Adjunctive use of corticosteroids is considered favorable, except in cases of fungal infection, or in immunocompromised individuals.

In this paper we try to obtain surgical treatment data from the most recent literature, based on different stages of infection. Several Authors have reported the importance of endoscopic surgery flanked by transcutaneous drainage with external approach and intraoral procedures, as shown in Table 2. We also reported the percentage of failures, according to the surgical procedure, in terms of disease recurrence, need for reoperation, and complications. The analysis of the literature reveals that the treatment of choice is Functional Endoscopic Sinus Surgery (FESS) which can be combined with a transcutaneous and/or intraoral approach in some specific cases.

**Table 2:** Our review of literature relative to the endoscopic surgery (FESS Functional Endoscopic Sinus Surgery) in the treatment of Orbital Cellulitis (OC), next to the failure rates of the procedures adopted reported by all authors

Author/Year	Type of study	No of cases	Treatment of orbital cellulitis	% of failures
Trimarchi et al. [5] 2019	Retrospective study	37	13 FESS 12 FESS + Dental procedure 9 FESS + Transoral 3 transcutaneous drainage	2.7% of total failure
Felisati et al. [10] 2013	Retrospective study	257	121 FESS 136 FESS + Transoral	0.8% 1.5%
Dewan et al. [11] 2011	Retrospective study	24	9 transcutaneous drainage 15 FESS + t.c drainage	55% No
Ikeda et al. [15] 2003	Retrospective study	10	4 FESS 6 transcutaneous drainage	No 16%
Pelton et al. [16] 2003	Retrospective study	11	1 FESS 4 transcutaneous drainage 6 FESS + t.c drainage	100% 25% No
Le et al. [18] 2014	Retrospective study	30	22 FESS 8 transcutaneous drainage	No 12.5
Nation et al. [19] 2017	Retrospective study	16	12 FESS 2 transcutaneous drainage 2 FESS + t.c drainage	No No No
Oxford et al. [20] 2006	Retrospective study	25	18 FESS 7 transcutaneous drainage	11.1% 14.2%
Rahbar et al. [21] 2001	Retrospective study	14	11 FESS 3 t.c drainage	18.2% No
Tabarino et al. [22] 2015	Retrospective study	20	11 FESS 9 transcutaneous drainage	No No
Tanna et al. [23] 2008	Retrospective study	13	11 FESS 2 transcutaneous drainage	54.5% 50%
Teinzer et al. [24] 2014	Retrospective study	37	31 FESS 6 FESS + t. c drainage	19% 16%
Yang et al. [25] 2009	Retrospective study	13	10 FESS 3 transcutaneous drainage	No No

In particular, Tsirouki et al. [13] suggested surgical treatment in presence of severe signs, such as compromised vision, pupillary changes, raised intraocular pressure, proptosis more than 5 mm, failure to respond to medical therapy, concurrent paranasal or frontal sinus infection, intracranial complications and clear dental source of the infection. Garcia et al. [14] recommended surgical procedure for dental infection because of the spread of anaerobes; the involved dental element can be treated by apicectomy or extraction. Felisati et al. [10] included transoral approach also to close oro-antral communications (OAC), to remove dislocated/failed implants, or to approach critical areas (i.e. maxillary alveolar recess, canine fossa, palatal region).

Dewan et al. [11] evaluated that pre-septal cellulitis does not require surgical therapy, while subperiosteal abscesses require it; successful transnasal endoscopic surgery is reported in patients with medial subperiosteal orbital abscesses, superolateral extension requires an external approach such as an incision over the superior orbital rim.

Ikeda et al. [15] suggested an external surgery also in subperiosteal abscess located in upper section medially in the orbit, through Lynch incision which offers adequate visibility and effective drainage but leaves more visible scars. In cases of limited view in medial abscesses in children, in addition to transnasal approach, Pelton et al. [16] suggested a transcaruncle approach, with better esthetic outcomes compared to the Lynch incision.

Torretta el al. [17] reported that endoscopic sinus surgery (ESS) has proved to be effective in treating SPA and IOA. Surgical steps include uncinectomy, middle meatal antrostomy, ethmoidectomy (usually anterior in SPA and anterior and posterior in IOA), and penetration of the *lamina papyracea*. This procedure seems to be appropriate in the case of medial locations (including those with a posterior extension).

In their review of the literature, Tsirouki et al. [12] proposed FESS approach for cases complicated with frontal sinusitis, and reserved evisceration only for patients who had fulminant orbital involvement, such as cases of bulbar perforation or endophthalmitis.

In cases with intracranial complications, surgical treatment is indicated and should be planned promptly after diagnosis: a delayed surgical drainage of brain abscesses is related to high morbidity and mortality. All patients with intracranial extension of the infection, require a combination of two or more surgical procedures: craniotomy, orbital surgery and sinus surgery.

In this study we reported three cases of patients suffering of odontogenic endo-orbital cellulitis. As soon as they arrived at our observation, we performed a careful clinical examination, a radiological study by CT and MRI (to evaluate the extent of the pathology) and an endonasal fibroscopy to evaluate the presence of purulent material inside the sinuses.

We started an empiric intravenous antibiotic treatment and then a therapy based on the antibiogram. In our cases it was necessary to combine antibiotic therapy and surgical treatment, both for the presence of orbital abscesses and for the odontogenic etiology. The surgical procedures that we performed were in agreement with the data obtained from the literature regarding surgical approaches that are summarized in Table 3.

**Table 3:** Review of literature about surgical approaches in the treatment of Orbital cellulitis (OC), Subperiosteal Abscess (SPA), Infraorbital Abscess (IOA)

Condition	Treatment
Medial OC/SPA/IOA - Medial with limited view - Medial in children	FESS (Functional endoscopic sinus surgery) OC/SPA: maxillary antrostomy + anterior ethmoidectomy OA: maxillary antrostomy + ant. and post. ethmoidectomy Combined FESS + Lynch incision Combined FESS + transcaruncular access
Inferior OC/SPA/IOA	FESS
Lateral OC/SPA/IOA	Lateral eyebrow incision with orbitotomy and drainage
Superior OC/SPA/IOA	Superior eyebrow incision with orbitotomy and drainage
Frontal sinus involvment	FESS
IOA with bulbar complications	Combined FESS + Evisceration
Dental infection (without implant dislocation or Oral antral communication OAC)	Combined FESS + extraction or endodontic treatment
Dental infection with: - Implant dislocation - OAC	Combined FESS +: - Transoral implant removal - Transoral OAC repair (eventually with local flap)

We used transnasal endoscopic approach based on maxillary antrostomy and ethmoidectomy (anterior in the first case and anterior and posterior in the second case) with infected dental element extraction. In the third case, evisceration was necessary to avoid intracranial spread, because an infraorbital abscess with bulbar perforation was already present, probably causing a spread of the infection through the cranial nerves and consequently severe intracranial complications. For the odontogenic etiology, we preferred to perform directly the extraction of the dental elements and not apicectomies, to avoid recurrence. Apart from tooth extraction, we avoided a transoral approach because in our three cases there was no evidence of OAC and dental or implant elements dislocated in the maxillary sinus.

Following these medical and surgical protocols, the three patients did not report any recurrence of the disease at follow-up.

## Conclusion

An early diagnosis is essential to implement a correct therapeutic plan and to avoid serious complications in odontogenic orbital cellulitis. Surgical treatment is quite simple but effective, especially in early-stage infections. It must be based on drainage of the orbital abscess and on removal of the odontogenic noxa. Surgical approach depends on the stages of the infection and on the site of involved structures: transnasal approach is preferred but in specific cases an external incision or an intraoral approach would be required.
